# Exploring SLEEPINESS through home monitoring with ultra-long-term subcutaneous EEG and ecological momentary assessment in sleepy treatment naïve obstructive sleep apnea patients starting CPAP treatment—A study protocol article

**DOI:** 10.3389/frsle.2024.1496923

**Published:** 2025-01-23

**Authors:** Mathias Sarkez-Knudsen, Martin Ballegaard, Henning Piilgaard, Esben Ahrens, Martin Christian Hemmsen, Tobias Søren Andersen, Jakob Eyvind Bradram, Preben Homøe

**Affiliations:** ^1^Department of Otorhinolaryngology, Zealand University Hospital, Køge, Denmark; ^2^Department of Neurology, Zealand University Hospital, Roskilde, Denmark; ^3^Department of Clinical Medicine, University of Copenhagen, Copenhagen, Denmark; ^4^T&W Engineering A/S, Allerød, Denmark; ^5^Department of Applied Mathematics and Computer Science, Technical University of Denmark, Lyngby, Denmark; ^6^Department of Health Technology, Technical University of Denmark, Lyngby, Denmark

**Keywords:** ultra long-term EEG, obstructive sleep apnea, sleepiness, home-monitoring, average sleep propensity, biomarker

## Abstract

**Introduction:**

Excessive daytime sleepiness (EDS) is a key symptom for patients with obstructive sleep apnea (OSA). Despite important limitations in the longitudinal monitoring of EDS, the Epworth Sleepiness Scale (ESS), the Maintenance of Wakefulness Test, and the Multiple Sleep Latency Test (MSLT) are the best available objective tests to predict EDS. Limited information exists on the day-to-day fluctuations of sleepiness symptoms from the everyday life perspective of OSA patients. The most feared is sudden sleep episodes that cause traffic accidents. The following study protocol investigates the novel possibilities of ultra-long-term Electroencephalography (EEG) (ULT-EEG) home monitoring in sleepy OSA patients with a subcutaneous implant. We hypothesize that the high-frequency testing from ULT-EEG, in combination with an ecological momentary assessment (EMA), can provide the information to develop new electrophysiological monitoring of sleep propensity as an alternative to the well-established, yet subjective, ESS.

**Methods:**

This clinical exploratory and experimental study will include 15 treatment-naïve patients with severe OSA, with a baseline ESS score above 10. The subjects will be implanted with a two-channel subcutaneous EEG monitoring device upon inclusion and a confirmative polysomnography MSLT. Subcutaneous EEG is recorded 24/7 for 6 weeks before and 6 weeks during continuous positive airway pressure (CPAP) treatment. Daily assessments with the Karolinska Sleepiness Scale, the Psychomotor Vigilance Task test, and a sleep/nap diary will be collected using EMA methods.

**Discussion:**

This study combines data collection from sleepy OSA patients' natural environments using ULT-EEG and EMA methods to obtain sleepiness metrics suitable for developing and preliminarily validating the possibilities of ULT-EEG sleepiness monitoring. We aim to prove a new concept of monitoring sleepiness symptoms in OSA patients and gain new insights into CPAP-related sleepiness rehabilitation.

**Ethics and dissemination:**

All participants will provide written informed consent to participate in this study. Ethical approval from the Region Zealand Ethics Committee on 13/09/2021 (SJ939, EMN-2021-06803). The study will be conducted in accordance with local legislation and institutional requirements and comply with the Declaration of Helsinki and the General Data Protection Regulation (GDPR).

## 1 Introduction

### 1.1 Background

Sleeping at the wheel is a feared complication for obstructive sleep apnea (OSA) patients living with excessive daytime sleepiness (EDS) (Higgins et al., 2017). Additionally, severely decreased quality of life and increased comorbidity and mortality are linked to EDS (Gooneratne et al., 2011). The Epworth Sleepiness Scale (ESS) is often used to clinically assess sleepiness in OSA patients, and it aims to determine average sleep propensity (Johns, 1991). The ESS constructs a standardized hypothetical test setting by asking the subject to estimate their risk of dozing off or falling asleep recently in eight everyday situations with different soporific characteristics. Yet, often, raters have not encountered these situations recently. Recall bias can either under- or overestimate the results, and the patient/doctor relationship plays an important role because the doctor gatekeeps specific solutions or restrictions that interfere with scoring the ESS, for example, the access to wake promoters in patients with residual EDS or situations in which driver-license suspension is at stake, to name some. Regarding the latter, L. Grote et al. have very clearly shown a double ESS score reduction for OSA patients starting CPAP treatment who were involved a driver's license certification process because of ongoing EDS even though the baseline ESS scores did not differ (Grote et al., 2018).

Objectively testing EDS in OSA patients is possible using the Multiple Sleep Latency Test (MSLT) and the Maintenance of Wakefulness Test (MWT). Determining the average sleep propensity using the MSLT, the MWT, or the ESS utilizes specific standardized conditions to determine the likelihood of falling asleep. This creates high reliability, but it poorly depicts the everyday context of sleepiness symptoms that often are far from these test conditions. These objective tests say much about how a subject performs in a specific test setting on a particular day, but translating that into an everyday setting is often a clinical challenge (Grote et al., 2018). Furthermore, the laboratory setting bypasses the patients' coping strategies for handling the EDS. The MSLT and the MWT are resource-intensive, and their utility as EDS-monitoring possibilities for the global OSA population, which is approaching 1 billion people (Pizza et al., 2013), is impossible.

In sleep medicine, the ability to use 24-h polysomnography (PSG) to predict MSLT results in the diagnostic workup of central hypersomnia has been shown (Pizza et al., 2013). A few attempts at recording Electroencephalography (EEG) as a measure of sleepiness in a subject's everyday life can be found in the scientific literature (Möller et al., 2009).

As sleep pressure regulation is a longitudinal phenomenon controlled by homeostatic and circadian processes, this phenomenon must be continuously monitored to optimally assess hour-to-hour, day-to-day, and week-to-week changes.

Microsleep (MS) episodes (Skorucak et al., 2020a; Moller et al., 2006) and frequency changes with increased theta power and decreased alpha power (Strijkstra et al., 2003) are some of the EEG-derived sleepiness markers suitable for a more hour-to-hour or day-to-day monitoring ability and have been found to correlate to performance measures (Moller et al., 2006).

In recent decades, technological advancements have brought forth devices capable of continuous EEG monitoring for weeks and up to years (Möller et al., 2009). One such device, the 24/7 EEG™ SubQ, can obtain good-quality and clinically relevant EEG data in this ultra-long-term perspective (Weisdorf et al., 2019). This offers a previously inaccessible great longitudinal dispersion but limited spatial resolution compared to standard EEG or the PSG mock-up, which has traditionally been limited to day(s)-long data recording. Recent studies found that despite the limited spatial resolution of ULT-EEG, automated EEG analysis for sleep–wake characterization performs well (Gangstad et al., 2019; Weisdorf et al., 2018).

The American Academy of Sleep Medicine (AASM) defines EDS as “the inability to maintain wakefulness and alertness during the major waking episodes of the day, with sleep occurring unintentionally or at inappropriate times almost daily for at least 3 months” (Lukacs and Bhadra, 2014). Likewise, the ESS asks the subject to estimate the risk of dozing off in “recent times”. With ULT-EEG recording systems, obtaining consecutive EEG throughout “recent times” is now possible. According to EDS definitions, sleep occurs as episodic intrusions during daytime wakefulness. This phenomenon is potentially measurable and, therefore, quantifiable. We hypothesize that this possibility will enable us to objectively measure what the ESS asks the patient to estimate.

### 1.2 Rationale for conducting this study

To the best of our knowledge, no previous study has been conducted recording ULT-EEG from OSA patients suffering from EDS to detect the degree and distribution (weekly/daily) of sleep phenomena in the patient's everyday life and during normal wake hours. In addition, limited knowledge is found on the effects of CPAP treatment on the rehabilitation from EDS from the perspective of day-to-day and week-to-week variations and CPAP therapy time.

There are many implications for the documentation and quantification of such sleep phenomena:

- A biomarker of OSA disease severity and CPAP treatment efficacy, as well as any other treatment attempts to reduce EDS.
- A clinical tool in the evaluation of fitness to drive and safety assessments regarding the sleepy OSA patient.
- Select OSA patients suffering from CPAP treatment-resistant residual sleepiness to guide indication of add-on wake promoters (e.g., solriamfetol).

### 1.3 Hypotheses

We hypothesize that at-home ULT-EEG monitoring can be used to monitor brain activity to the extent that quantification of sleep intrusions in the daytime can be detected in the natural environment of patients suffering from EDS due to OSA.

This suggests a potential new methodology for quantifying physiological sleep pressure or average sleep propensity derived directly from the patient's natural environment, taking into consideration patient-specific coping and other behaviors.

We believe that ULT-EEG measured at home holds novel information to bring forth biomarkers suitable for OSA patients and aid in better treatment-effectiveness monitoring and safety assessments. This study will serve as a proof of concept for using ULT-EEG monitoring when evaluating EDS in OSA patients.

### 1.4 Objectives

This project aims to develop new sleepiness biomarkers using ultra-long-term at-home EEG monitoring as a novel, new data type. We will conduct a clinical study exploring electrophysiological sleep/wake transition phenomena in a population with OSA and EDS by making ultra-long-term subcutaneous EEG recordings in subjects' real-life conditions before treatment and as they start treatment.

We follow an explorative objective and an experimental one. We aim to validate our explorative findings in an experimental setting.

Our *explorative* objective is to scrutinize subcutaneous ULT-EEG for potential biomarkers predicting sleepiness in OSA patients. Determining daytime wake-to-sleep transition rate, microsleep episodes, and macro-sleep episodes, alongside the duration and patterns of these events. In addition, vigilance determination of the odds ratio product (ORP) (Younes, 2023) or Vigilance Algorithm Leipzig (VIGALL) (Olbrich et al., 2015) can be assessed. Our explorative findings will be correlated and preliminarily validated against the well-established sleepiness metrics and questionnaires, the MSLT, the ESS, the Karolinska Sleepiness Scale (KSS), and the Psychomotor Vigilance Task (PVT) test, as well as patients' experiences of falling asleep or dozing off in the form of a “sleep and nap diary”. Any potential sleepiness biomarker derived from this study will be available for future confirmatory trials.

Our *experimental* objective is to determine the effects of CPAP treatment on sleep quality, quantity, and timing from the day-to-day perspective that ULT-EEG provides. Furthermore, the experimental design enables us to clinically validate the exploratively deduced sleepiness biomarker candidates through comparisons before and after the intervention of CPAP treatment conditions. Previous studies on CPAP's effect on reducing sleepiness have shown a dose-dependent configuration with CPAP usage. ESS scores are typically reduced by 4.75 points (Patel et al., 2003). We expect relevant biomarker candidates to follow similar patterns when CPAP is introduced in a population with severe OSA and EDS.

Our *secondary objective* is to validate automatic sleep/wake classification based on two-channel subcutaneous EEG against golden standard manually scored polysomnography in patients with severe OSA. Additionally, we explore using ecological momentary assessment (EMA) methods to record excessive daytime sleepiness in patients with OSA and collect passive sensor data from participants' smartphones to assess physical activity.

### 1.5 Study design

This study is a clinical prospective exploratory and experimental study of patients suffering from EDS because of severe OSA.

**Figure 1:**
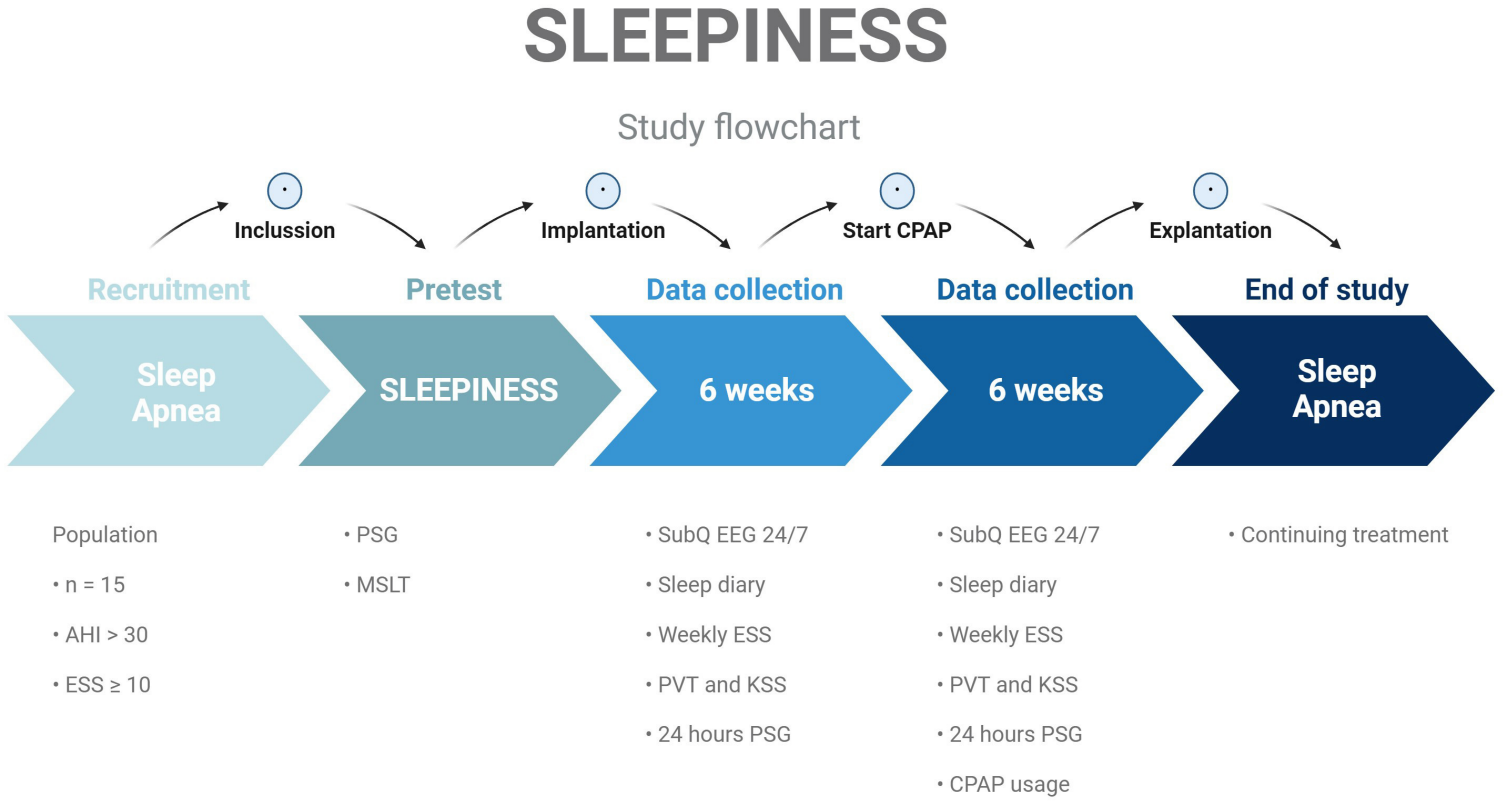
Patients screened according to predefined inclusion/exclusion criteria and found eligible and willing to participate will follow a timeline of PSG and MSLT. Implantation of 24/7 SubQ EEG device. Six weeks recording of subcutaneous EEG, daily sleep diary and episodes of daytime sleeping, weekly ESS scores, and daily KSS scores and PVT scores. Start CPAP treatment. Six weeks of recording with a collection of CPAP usage data. Explanation of 24/7 SubQ EEG device. End of study. AHI, Apnea-Hypopnea Index; ESS, Epworth sleepiness Scale; PSG, polysomnography; MSLT, Multiple Sleep Latency Test; PVT, Psychomotor Vigilance Task test; KSS, Karolinska Sleepiness Scale; CPAP, continuous positive airway pressure.

We include 15 treatment naïve severe sleep apnea patients with an ESS score above 10. See Figure 1 for an overview of the study flow. The subjects enter the study as new referrals to the sleep clinic at Zealand University Hospital, Denmark, and will, after inclusion, start the study with a pretest with a PSG, followed by an MSLT. If these test results conflict with the inclusion and exclusion criteria the subject will be excluded from the further study.

The subjects will have the UNEEG™ SubQ implanted (24-7 EEG™ SubQ”), and after approximately 10 days of healing, they will start wearing the external recorder as much as possible for the following 12 weeks. CPAP treatment is started 6 weeks after the start of ULT-EEG recording. Two secondary PSGs will be performed after implantation: the first prior to OSA treatment initiation and the second after. During the ULT-EEG recording, simultaneous recording of weekly ESS scores and a daily sleep diary will be performed through the participants' smartphones. Additionally, 14 days before and during CPAP treatment, the KSS and PVT test will be performed four times daily. For further information on these assessments, see Section 2.4 “Study Procedures.”

### 1.6 Study endpoints

• ULT-EEG is analyzed to assess potential electrophysiological biomarkers resembling sleepiness or sleep pressure, such as wake–sleep transition rate, timing, and daytime sleep patterns in the micro and macro perspective or vigilance state classification. This will be assessed through automated microsleep detection and automated sleep-stage classification methods. Using concomitant PSG and ULT-EEG, we will identify the markers of stage transition from awake to dozing/light sleep in the SubQ recording. We will do this both according to AASM criteria and in shorter segments of EEG. From this template, we will use machine learning (ML) techniques to train a classifier to detect those short transitions in the ULT-EEG recording with different levels of sensitivity and specificity.

• The correlation between ULT-EEG-derived biomarker candidates and well-known sleepiness metrics, such as the mean sleep latency and EMA collected reaction time, and ESS and KSS scores, as well as patient reports of episodes of daytime sleeping or dozing off (nap diary). From the timestamps on PVT prompts to patients, we will identify segments of interest in the ULT-EEG recording to document changes in EEG-related signs of reduced vigilance. We will use this to validate the responses on the clinical scales given by patients.

## 2 Method and material

### 2.1 Participants and study setting

Subjects eligible for this study are patients who suffer from EDS because of untreated OSA and those meeting the inclusion/exclusion criteria listed later to ensure the generalizability of the study and minimize the influence of other contributors to EDS. The study will take place at Zealand University Hospital, Denmark. Study subjects will not receive any compensation for their participation.

### 2.2 Intervention

The clinical intervention in this study is the start of treatment with nocturnal continuous positive airway pressure (CPAP). CPAP treatment is the gold standard for moderate and severe OSA treatment and works by opening the upper airway through increased upper airway pressure. CPAP compliance has typically been set at least 4 h each night, 70% of nights, even though this line is arbitrary. CPAP is very effective at reducing Apnea-Hypopnea Index (AHI) scores but has not shown an effect in randomized placebo-controlled trials on endpoints like new cardiovascular events. However, for the effect of CPAP on EDS reduction, a “dose-dependent” effect is described in two studies (Weaver et al., 2007; Antic et al., 2011).

Assessing ULT-EEG in the 6 weeks leading up to CPAP initiation as well as for 6 weeks on treatment gives us the opportunity to compare a treatment-naïve state with a period on treatment, thereby determining the effect of CPAP on ULT-EEG and EMA-assessed sleepiness metrics. Previous studies on patients with severe OSA and baseline ESS scores above 10 have found CPAP treatment to reduce the ESS score by 4.75 points (Patel et al., 2003). Within 6 weeks of CPAP treatment, we expect to see an initial and partial sleep rehabilitation effect, as other studies have found that sleep remained disturbed as assessed by continuous actigraphy 3 months after CPAP initiation (Tippin et al., 2016).

### 2.3 EEG recording device for ultra-long-term EEG recordings

**Figure 2:**
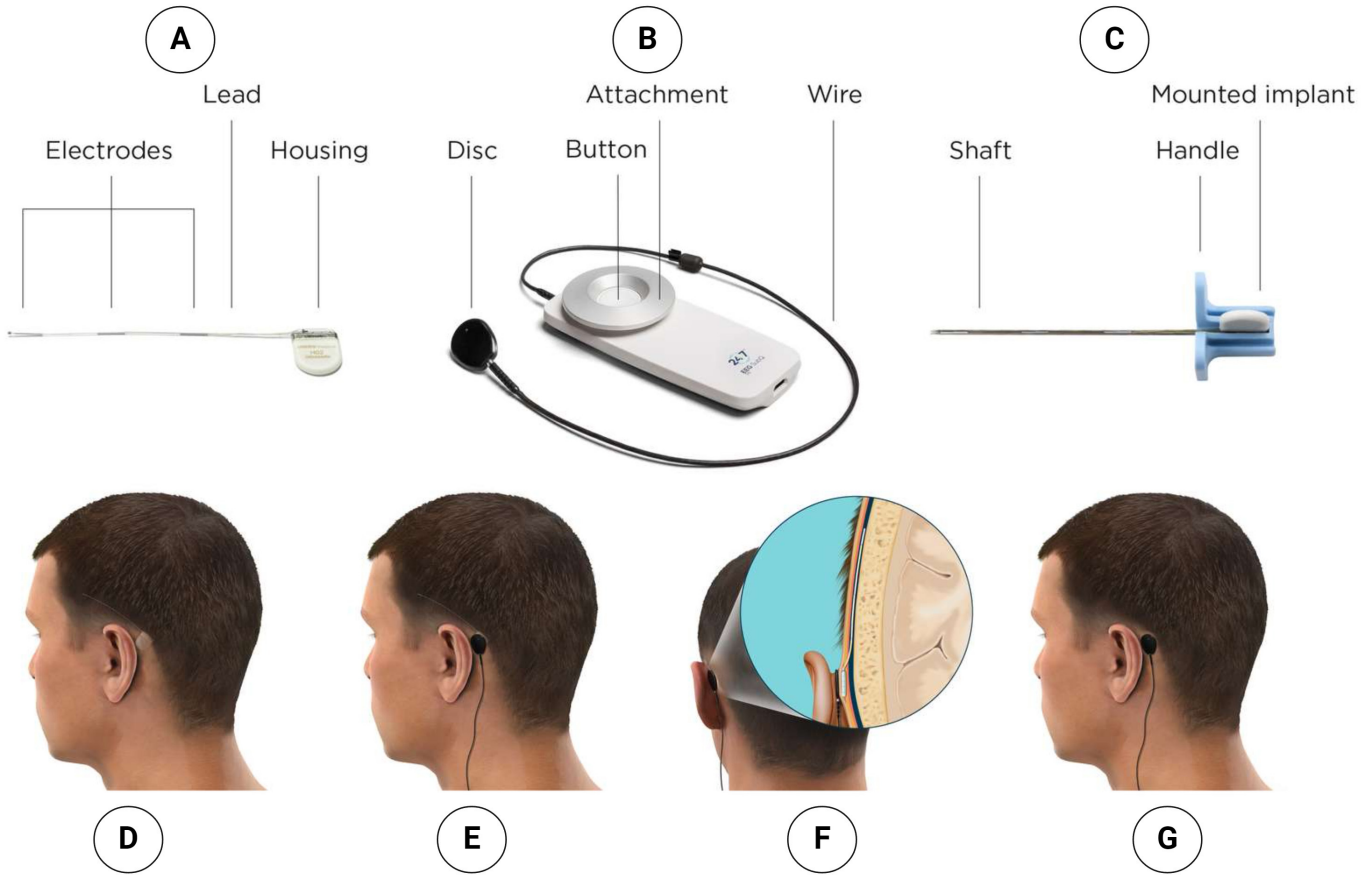
**(A)** Implant. **(B)** Recorder. **(C)** Introducing needle with mounted implant. **(D)** Implant is placed subcutaneously to collect EEG data. **(E)** External recorder attached to the skin behind the ear, placed directly above the implant housing. **(F)** Implant housing is placed subcutaneously behind the ear; lead with three electrodes is placed in the subgaleal space. **(G)** Small and discrete solution collects EEG data day and night during everyday life.

**Figure 3:**
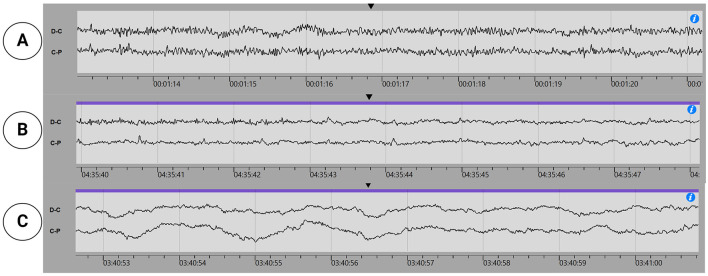
Subcutaneous EEG snippets: **(A)** Wake, **(B)** Non-REM sleep stage 2, and **(C)** Non-REM sleep stage 3.

The ultra-long-term (>2 weeks) EEG recordings will be made using the 24/7 EEG™ SubQ, a Conformité Européenne (CE)-approved device manufactured by UNEEG™ Medical A/S, Borupvang 2, 3450 Allerød, Denmark. See Figure 2 for an image of the implant, recorder, introducing needle, and the device setup for the subjects. The system is a two-channel EEG recorder device (Viana et al., 2021) and has mostly been utilized in long-term epilepsy monitoring (Grewe et al., 2020). By double-tapping the button on the recorder, the participant can record an event in the EEG signal suitable for subjective attack registration. In experienced hands, the implantation or explantation of the device can be performed in 15 min, and the device is well tolerated by most people (Djurhuus et al., 2023). Recently, its utility in ultra-long-term sleep determination has been documented (Ahrens et al., 2023, 2024a), and stable signal quality in the ultra-long-term perspective (weeks) for spectral analysis, thereby making study design containing intervention possible (Viana et al., 2021). Despite the limited spatial resolution of two EEG channels, Gangsted et al. reached a sensitivity of 94.8% and a specificity of 96.6% using the device to detect sleep vs. wake in patients with epilepsy (Gangstad et al., 2019). See Figure 3 for an example of EEG signals recorded by the SubQ device resembling wakefulness, non-Rapid eye movement (non-REM) sleep stage 2, and non-REM sleep stage 3.

### 2.4 Study procedures

#### 2.4.1 The implantation

The 24/7 EEG SubQ electrode will be implanted behind the ear, with the electrode positioned over the temporal lobe. The side chosen is the one the patient least sleeps on. The subjects will be instructed in mounting and using 24/7 EEG™ SubQ (according to the instruction for use) as much as possible during sleep and daytime in the study period. The subjects will be asked to create an event by double-tapping the power button on the logger when they turn off the lights and go to sleep or if they experience an episode of unintentional sleep during the day.

#### 2.4.2 PSG and MSLT

The PSG will be mounted by trained personnel on a study site and performed according to the AASM criteria for the PSG mock-up. The PSG night recordings will be made in an outpatient setting where the subjects sleep in their homes. The following day the subjects will meet on-site for the MSLT. The MSLT study protocol will follow the AASM guidelines. After that, the PSG equipment will be demounted. The PSG/MSLT recordings will be performed using CE-approved equipment. The PSG and MSLT data analysis will be conducted by a trained sleep technician according to the AASM criteria and evaluated by a physician with expertise in sleep medicine. The clinically relevant results of the PSG and MSLT will be given to the subject by a medical doctor during the following on-site visit.

#### 2.4.3 Interview

During the study period, the study subjects will be invited to participate in semistructured interviews focused on sleepiness symptoms and everyday life before and after treatment initiation.

#### 2.4.4 EMA and mobile sensing

EMA is a method for assessing patient-reported outcome measures (PROMs) in the actual situation being assessed. PROMs are always reported in a context in which the reporting might interact. In EMA studies, subjects can be tested in the context they are actually in, such as at home. EMAs are typically collected using a smartphone app installed on participants' phones. This approach has great advantages over paper and pencil, including reducing errors, avoiding “backfilling” of the sleep diary, and providing digital timestamps with exact time registrations for when the subject completed their PVT test and KSS. Furthermore, participants have been shown to have their smartphones with them at all times, including nighttime, which makes it a well-suited platform for home-based sleep studies.

This study collects subjective sleepiness scores, PVT test scores, and sleep diary data by prompting subjects to register these on their smartphones. The active collection of EMA data is supplemented with simultaneous passive mobile sensing, collecting GPS-determined location, step counts, activities detected by the phone (e.g., walking, running, and biking), screen events, battery time, and local weather data. This set of passive sensing data helps build a rich contextual picture of participants' activity levels and temperature exposure during the study. These factors are all relevant to sleepiness perception in the everyday setting. In determining sleepiness levels, it is evident that it is a situational phenomenon, and clinical variability, for instance, ESS scores, has been reported with significant day-to-day or within-day variance that was probably explained by situational sleepiness (Grewe et al., 2020).

In this study, we use the Copenhagen Research Platform (CARP) and the CARP Mobile Sensing Framework (Bardram, 2022, 2020) to collect the active EMA PROMs and the passive sensing data. CARP is an open-source research tool designed for digital phenotyping, accessible on both Android and iPhone (iOS) smartphones. In this study, data will be collected by the Technical University of Denmark (DTU) by hosting an instance of CARP and ensuring compliance with GDPR and information security. The CARP framework supports in-app consent procedures, the triggering of EMA surveys and tasks, and the automatic collection of passive sensor data.

#### 2.4.5 Subjective sleepiness and alertness

The KSS will be used to determine sleepiness levels at different times during the day. The KSS is a sleepiness rating scale from 1 (*very alert*) to 9 (*very sleepy, fighting sleep, and effort to keep awake*) We utilize the “state” dimension of the KSS in opposition to the “trait” dimension of the ESS. A 3-minute reaction-time test called the PVT test is used for alertness determination. The PVT test depicts the neurobehavioral effect of sleep loss or circadian misalignment, which is considered the gold standard. In this study, the KSS and the PVT test are EMAs, and the subjects will be prompted on their smartphone to fill these out four times a day during the 14 days before and 14 days after CPAP initiation. The specific prompting time will be at 08:00, 12:00, 16:00, and 20:00. In addition, the KSS and PVT EMAs will be available for the subject for an “at any time” registration. The subjects are instructed to take the test whenever they experience high subjective sleepiness episodes. Using this method, we bypass the global retrospective self-report necessary for filling out, for example, the ESS, which can be affected by recall bias.

#### 2.4.6 Sleep and nap diary

During the 12-week study, the participant will fill in daily “sleep and nap diary”, subjective registration of napping patterns, and sudden sleep episodes. At 08:00, the participant will receive a notification to fill in a sleep survey, asking, “What time did you get out of bed today?” and “When did you go to sleep last night?” At 21:00, the participant will receive a notification to register sleep or nap episodes that might have occurred during the day, asking, “Have you been sleeping during the day?” If yes, an additional question is posed: “How long have you been sleeping during the day?” For every daytime sleep episode, the participant is asked to determine the type of nap: “I consciously took a nap because I felt the need to”, “I fell asleep without the intention to”, and “I registered I had fallen asleep when I suddenly woke up”. The subjects are asked to press the event button of the 24/7 SubQ device one time if they experience waking up from an unintended nap during the daytime. Likewise, the subjects are asked to press the event button twice before taking a planned nap during the day. This records an event in the EEG signal.

### 2.5 Inclusion criteria

For a subject to be eligible, all inclusion criteria must be answered “yes”:

1. Age ≥18 years.
2. Must be diagnosed with severe OSA (AHI score >30) and have not yet started any form of positive airway pressure treatment.
3. ESS score above 10 at the time of inclusion.

### 2.6 Exclusion criteria

For a subject to be eligible, participants must answer “no” to all exclusion criteria:

1. Known severe neurological or psychiatric diseases.
2. Known congestive heart failure, chronic renal failure, liver failure, malignancy, or severe pulmonary disease.
3. Considerable use of alcohol.
4. Medication judged by the investigator to influence sleep/wake regulation to such a degree that data quality will be compromised.
5. Has cochlear implants.
6. Involved in therapies with medical devices that deliver electrical energy into the area around the implant.
7. Is at high risk of surgical complications, such as active systemic infection and hemorrhagic disease.
8. Are unable (i.e., mentally or physically impaired patient) or do not have the necessary assistance to operate the device system properly.
9. Has an infection at the site of device implantation.
10. Operates magnetic resonance imaging scanners.
11. Has a profession/hobby that includes activity imposing extreme pressure variations (e.g., diving or parachute jumping). Nota Bene (NB): diving/snorkeling is allowed to a 5-meter depth.
12. Has a profession/hobby that includes activity imposing an unacceptable risk for trauma against the device or the site of implantation (e.g., martial arts or boxing).
13. Other known diseases or conditions judged by the investigator to influence sleep to such a degree that data quality will be compromised.
14. Incapable, judged by the investigator, of understanding the participant instructions or is not capable of carrying through with the investigation.
15. Pregnancy or intention to become pregnant within the next 12 months.

### 2.7 Statistical considerations

As this is an exploratory and observational study, a similar exploratory approach will be used for statistical analysis. Therefore, the statistical analysis plan is not fixed, and the choice of statistical methods will be data-dependent. Data will be scrutinized for potential biomarkers related to EDS. The data analysis will be conducted with the DTU. The preliminary approach is based on classical time domain and frequency domain analysis. Artificial intelligence and ML will be used when applicable.

We aim to identify sleep/wake regulation patterns through several methodological approaches like VIGALL, microsleep detection, and macro-sleep/napping detection and correlate these to well-established metrics of sleepiness, such as mean sleep latency, reaction time, subjective sleepiness scores, and previous night's metrics of sleep quality.

### 2.8 Power analysis

We use the ESS score to guide power analysis for this exploratory study. The ESS score is the typically used clinical tool for assessing EDS in OSA patients. A meta-analysis on the effect of CPAP on patients with baseline ESS >10 found a mean pooled average ESS score reduction of 4.75 (95% CI [2.97, 6.53], *p* < 0.001) compared with those in the control group (Patel et al., 2003). As we explore new biomarker candidates, we aim to be able to correlate/validate these against a well-known clinical effect. We anticipate the effect size of CPAP on ESS reduction to be of a moderate or large difference effect (>0.3). The ESS score is measured once weekly for 6 weeks before starting CPAP and six times while on CPAP treatment. In addition, the baseline ESS score is measured at inclusion. Using the G^*^Power3.1.9.7 software and repeated-measures within-factor analysis of variance, we calculate a total sample size of 12 participants that would ensure a power of 95%. Previous studies in sleepiness quantification have shown considerable intra-individual variability (Skorucak et al., 2020b). Because of missing data or dropout, we aim to include 15 subjects.

### 2.9 Methodological consideration on monitoring sleepiness through ULT-EEG

#### 2.9.1 Assessing sleepiness through the lens of ULT-EEG

Several methodological approaches can identify sleepiness through sleep–wake regulation instability. Here, we present three: daytime macro-sleep pattern identification, daytime MS pattern identification, and EEG-vigilance identification.

#### 2.9.2 Macro daytime sleep detection

DeepSleepNET, a model for automatic sleep-stage scoring based on raw single-channel EEG (Supratak et al., 2017), and the U-sleep model by Perslev et al. (2021) are open-source automated sleep-scoring algorithms that can be used to automatically determine a classical hypnogram on PSG data. Fine-tuning this model will determine sleep events during the daytime in patients' everyday lives instead of asking subjects to determine their likelihood of dozing off in eight everyday situations, as is the case of the ESS (Ahrens et al., 2024b).

#### 2.9.3 MS detection during the daytime

Despite EEG signals having a very good temporal resolution the historical tradition of sleep/wake scoring is still based on the 30-s-epoch approach. This tradition originates from the early days of EEG recording when a 30-s EEG signal would fit a normal paper size. Every paper/30-s epoch of EEG recording was then, and is still today, assigned to one sleep stage according to what most of the EEG signal resembles. Thereby, information is lost, and the very good temporal resolution of the EEG signal is tremendously reduced. One way to regain the lost information is to score sleep in timeframes shorter than the classical 30-s frame. This is called MS and is an episode of EEG with sleep characteristics that lasts < 15 s. In healthy adults, microsleep events during the day accumulate during a 7-day sleep restriction protocol (Bougard et al., 2018). In addition, even though the MSLT is the most validated objective measure of sleepiness, V. Tirunahari and colleagues (Tirunahari et al., 2003) found that addressing MS determination during the MSLT is a more specific and sensitive test for EDS compared to the MSLT on its own. Therefore, MS detection during daytime is a potentially objective measure of daytime sleepiness. Varying definitions of MS events have been suggested in the literature, and the Bern University Hospital's continuous and high-resolution wake-sleep (BERN) scoring criteria resemble a recent set of standardized criteria (Hertig-Godeschalk et al., 2020). MS determination in OSA patients (Morrone et al., 2020), as well as healthy adults (Bougard et al., 2018), is reported to be a marker of sleep pressure. In addition, a correlation was found between mean MS incidence and crash risk in driving simulation (Moller et al., 2006). Reliable MS event (MSE) detection is possible with an automated approach like machine learning/deep learning (Skorucak et al., 2020a; Malafeev et al., 2021). A recent publication on MS during maintenance of wakefulness test found two distinctive patterns of MSE in which a series of multiple MSE correlated to shorter sleep latency, indicating a higher degree of sleepiness, whereas a single isolated MSE correlated to longer sleep latency or even no sleep during an MWT. The study further illuminates the borderland between wakefulness and sleep by describing that for most patients, two or more MSEs were found before manifest sleep occurs in the MWT and the time from the first MSE to sleep onset varied considerably across the population (Hertig-Godeschalk et al., 2020).

#### 2.9.4 Vigilance detection

Another way of addressing sleepiness is through the evaluation of vigilance. Based on a 15-minute resting EEG, the automated VIGALL method (Olbrich et al., 2015) categorizes EEG-vigilance regulation as stable, medium, or unstable. Every 1-s EEG segment is classified into a certain set of vigilance stages that range from 1 to 7. Furthermore, using the continuous numeric measure the ORP to determine vigilance from EEG is possible (Younes, 2023).

## 3 Discussion

The novelty of this protocol lies in its methodology of assessing sleepiness phenomena in patients' natural environment. This is an interesting new modality for quantifying sleepiness or sleep pressure for OSA patients, but this approach seeks discussion in the scientific area of interest.

Sleepiness assessments derived from ULT-EEG and EMA from “real life” bypass patients' need to recall the degree of sleepiness and patients' motivation for highlighting or diminishing a specific symptom. Furthermore, it takes into account the subject-specific coping strategies in handling sleepiness symptoms, for example, planned napping before monotonous or risk-related tasks or a voluntary increase in external alerting factors.

Utilizing the combination of ULT-EEG monitoring alongside EMA methods and automated data analytical breakthroughs seems a promising new arena for the sleep/wake interplay evaluation of patients with excessive daytime sleepiness. These possibilities enable us to concomitantly assess the sleepiness phenomenon from the three dimensions Baiardi and Mondini (2020) describe: introspective, physiological, and manifest all at once. This data triangulation is in our protocol shown by ESS and KSS scores, ULT-EEG, and PVT scores and provides an interesting platform for evaluating EDS in OSA patients.

But, from a phenomenological point of view, we are aware of the terminological dissonance between documenting MS events and the subjective experience of sleepiness or loss of alertness perceived as sleep pressure or need to sleep. Parallel to that, recent vigilance research has challenged the traditional view of sleep and wakefulness as mutually exclusive states, suggesting sleep is both local and complex. Studies using intracranial EEG and other advanced techniques reveal that sleep-like activity can occur in specific brain regions during wakefulness (Andrillon and Oudiette, 2023; D'Ambrosio et al., 2019). This local sleep has been associated with attentional lapses and cognitive impairments (Andrillon et al., 2019). Sleep onset and intensity vary across brain areas, with frontal regions often showing earlier and deeper sleep (Ferrara and de Gennaro, 2011). Slow waves and sleep spindles, key features of non-REM sleep, can occur locally and out of phase in different brain regions (Nir et al., 2011). Despite this local regulation, a global sleep mechanism likely exists to ensure behavioral shutdown for safety and efficiency (Rattenborg et al., 2012). This evolving understanding of sleep and wakefulness as both global and local brain processes, influenced by various physiological systems, has implications for sleep phenomena assessed by a unilateral EEG implant such as presented in this protocol.

### 3.1 Discrepancies between subjective sleepiness, behavioral factors, and dozing-off phenomena

Making objective measures and quantifying the multifaceted phenomenon of sleepiness has been referred to as the holy grail of sleep medicine (Bliwise, 2001). This is probably because several factors, such as physiological, psycho/social, and behavioral factors, influence sleepiness. We look into the differences between dozing off and reporting feeling sleepy, as we find that these concepts are not interchangeable (Thorarinsdottir et al., 2019). Being capable of feeling sleepiness is suggested as a protection mechanism to help restrain risk-related activities, such as car driving, because of the potential reduced alertness (Cai et al., 2021). This mechanism is likely altered differently across the different origins of sleepiness, such as Parkinson's disease, narcolepsy, or OSA (Möller et al., 2009). For example, “blank spells” or sudden onset sleep without prior sleepiness perception have been described for Parkinson's patients but not for OSA patients (Hobson et al., 2002).

### 3.2 Additional possibilities from the ultra-long-term perspective

Even though the pathophysiological mechanisms linking EDS and OSA are primarily considered to be intermittent hypoxemia and sleep fragmentation, a recent systematic review argues that circadian misalignment caused by OSA also plays an important role in the genesis of EDS (Šmon et al., 2023). Sleep/wake registration with ULT-EEG might provide us with new information on the circadian misalignment of OSA patients and the effect of CPAP initiation.

ULT-EEG not only has the potential to determine daytime markers of sleep pressure, but it also contains the possibility to correlate sleep pressure to the previous day's sleep quality metrics, such as total sleep time, degree of deep sleep, or arousal index. With automated sleep algorithms gaining near-human robustness in sleep classification, it is not wishful thinking that OSA patients might be provided real-time feedback on which adjustable sleep parameters affect their sleepiness like a biofeedback system.

## 4 Outcome and impact

OSA patients have been shown to have an increased risk of motor vehicle and work-related accidents (Udholm et al., 2022; Garbarino, 2020; Gharibi et al., 2020). Many measures have been tried to improve driver drowsiness detection (Higgins et al., 2017). Because sleepiness-related accidents are potentially preventable, we are obligated to improve our evaluation of fitness to drive to reduce the incidence of future accidents. To do so, we need to get more specific than “average sleep propensity” in the objective measure of EDS in OSA patients often assessed by the ESS, especially when ESS scores have been found to vary considerably within individual patients (Walker et al., 2020). The possibility of providing patients feedback on sleepiness fluctuations from their everyday lives might be a powerful tool for coping with, handling, and mitigating the accident risk of sleepiness for OSA patients and beyond.

In this study, we will use an implantable ULT-EEG device, but we acknowledge that such devices are unsuitable for the general OSA population. We hope our findings will be transferable to non-invasive and unobtrusive wearables like long-term EEG assessed in the ear canal (Kappel et al., 2019).

In Johns (1991) published an article titled “A New Method for Measuring Daytime Sleepiness: The Epworth Sleepiness Scale”. Through this SLEEPINESS project, we aim to have history repeat itself by developing a novel, new way of measuring sleepiness. What Holter monitoring is to cardiology, ULT-EEG might be to the field of sleep medicine.

## 5 Limitation

Light sleep (stage N1) determination is typically the most difficult sleep stage to distinguish from the others. As we aim to detect sleep in two-channel subcutaneous EEG during periods of expected wakefulness, performance measures on N1 stage determination are of utmost importance.

Sleepiness determination through ULT-EEG lacks normative data, and how the ULT-EEG-found levels of sleepiness correlate to physiological vigilance, decision-making, or driving performance is unknown. This must be validated in future confirmatory trials.

Due to safety measures, OSA patients with driver's license suspension upon referral, typically ESS score >15, are not included in this project. This limits the population to those with ESS scores between 10 and 15. Hence, our population will not have severely increased ESS scores >15.

We assess a pre-CPAP treatment phase with the hope of gaining an understanding of living with EDS because of untreated OSA for the many yet-undiagnosed OSA patients, but our pre-CPAP treatment data are biased because our subjects will have disease-specific knowledge that can change behaviors like sleep patterns or lifestyle.
