# Supplementary material for: Exploring SLEEPINESS through home monitoring with ultra-long-term subcutaneous EEG and ecological momentary assessment in sleepy treatment naïve obstructive sleep apnea patients starting CPAP treatment—A study protocol article
Source: Front Sleep. 2025 Jan 23;3:1496923. doi: 10.3389/frsle.2024.1496923 (PMC12713844; doi:10.3389/frsle.2024.1496923)
Supplement: Supplementary file 1 [file Table_1.docx]

Appendix 1 – List of Abbreviations

AASM: American Academy of Sleep Medicine

AHI: Apnea-Hypopnea Index

AI: Artificial Intelligence

ASP: Average Sleep Propensity

BERN criteria: Bern University Hospital's continuous and high-resolution wake-sleep criteria

CARP: Copenhagen Research Platform

CPAP: Continuous Positive Airway Pressure

DTU: Technical University of Denmark

EDS: Excessive Daytime Sleepiness

EEG: Electroencephalography

EMA: Ecological Momentary Assessment

ESS: Epworth Sleepiness Scale

KSS: Karolinska Sleepiness Scale

ML: Machine Learning

MSE: Micro Sleep Event

MS: Microsleep

MSLT: Multiple Sleep Latency Test

MWT: Maintenance of Wakefulness Test

ORP: Odds Ratio Product

OSA: Obstructive Sleep Apnea

PROM: Patient-Reported Outcome Measures

PSG: Polysomnography

PVT: Psychomotor Vigilance task Test

ULT-EEG: Ultra-Long-Term EEG

VIGALL: Vigilance Algorithm Leipzig
